# A cross-sectional study of cognitive performance in bipolar disorder across the lifespan: the cog-BD project

**DOI:** 10.1017/S0033291722003622

**Published:** 2023-10

**Authors:** Brett D. M. Jones, Brisa S. Fernandes, M. Ishrat Husain, Abigail Ortiz, Tarek K. Rajji, Daniel M. Blumberger, Meryl A. Butters, Ariel G. Gildengers, Tatiana Shablinski, Aristotle Voineskos, Benoit H. Mulsant

**Affiliations:** 1 Department of Psychiatry, Temerty Faculty of Medicine, University of Toronto, Toronto, ON, Canada; 2 Campbell Family Research Institute, Centre for Addiction and Mental Health, Toronto, ON, Canada; 3 Toronto Dementia Research Alliance, Temerty Faculty of Medicine, University of Toronto, Toronto, ON, Canada; 4 Department of Psychiatry, University of Pittsburgh, Pittsburgh, PA, USA

**Keywords:** Bipolar disorder, cognition, geriatric psychiatry, mood disorder

## Abstract

**Background:**

Neuroprogressive models of the trajectory of cognitive dysfunction in patients with bipolar disorder (BD) have been proposed. However, few studies have explored the relationships among clinical characteristics of BD, cognitive dysfunction, and aging.

**Methods:**

We conducted a cross-sectional analysis in euthymic participants with the MATRICS Cognitive Consensus Battery, the Trail Making Test B, the Stroop Test, and the Wechsler Test of Adult Reading. Age- and gender-equated control participants without a mental disorder [‘Healthy Controls’ – HC)] were assessed similarly. We compared cognitive performance both globally and in seven domains in four groups: younger BD (age ⩽49 years; *n* = 70), older BD (age ⩾50 years; *n* = 48), younger HC (*n* = 153), and older HC (*n* = 44). We also compared the BD and HC groups using age as a continuous measure. We controlled for relevant covariates and applied a Bonferroni correction.

**Results:**

Our results support both an early impairment (‘early hit’) model and an accelerated aging model: impairment in attention/vigilance, processing speed, and executive function/working memory were congruent with the accelerated aging hypothesis whereas impairment in verbal memory was congruent with an early impairment model. BD and HC participants exhibited similar age-related decline in reasoning/problem solving and visuospatial memory. There were no age- or diagnosis-related differences in social cognition.

**Conclusion:**

Our findings support that different cognitive domains are affected differently by BD and aging. Longitudinal studies are needed to explore trajectories of cognitive performance in BD across the lifespan.

## Introduction

Bipolar disorder (BD) is a severe mental illness that affects approximately 1–2% of the population (Grande, Berk, Birmaher, & Vieta, 2016). Most patients with BD experience cognitive impairments: several meta-analyses have reported impairments of medium to large effect size relative to healthy individuals in various cognitive domains, in particular, verbal memory impairment and executive dysfunction (Bourne et al., 2013). These impairments are pronounced during depressive episodes and have been shown to improve with resolution of depressive symptoms (van Rheenen et al., 2020); they are also highly prevalent during mania and persist during euthymia (Bora, Yucel, & Pantelis, 2009; Lopes & Fernandes, 2012). Persistence of cognitive dysfunction is one of the strongest predictors of impairment in psychosocial functioning (McIntyre, 2020) (van Rheenen et al., 2020). There is also evidence that older adults with BD (OABD) show worse performance in cognition across multiple domains, with the largest effect sizes for verbal learning and verbal/visual delayed memory (Montejo et al., 2022b). However, there is also evidence that cognitive dysfunction may be present at the time of the first episode and possibly precede the onset of mood symptoms (Martino, Samamé, Ibañez, & Strejilevich, 2015).

While there is evidence of cognitive dysfunction in both younger and older persons with BD, only a few studies have investigated the trajectory of cognition in BD longitudinally. In a meta-analysis of 12 such studies, performance on 14 cognitive measures remained stable over a mean follow-up period of 4.6 years (Samamé, Martino, & Strejilevich, 2014). Similarly, in another meta-analysis, there were no cognitive changes in BD patients over either the short-term (1.5 years) or longer-term (5.5 years) (Bora & Özerdem, 2017). In a three-year cohort study of patients with a first episode of mania and control participants, patients with BD presented with cognitive deficits compared to controls but both groups had a similar trajectory (Torres et al., 2020). However, in another study, OABD showed a steeper decline in executive function than older controls (Seelye et al., 2019).

Even fewer studies have focused on cognitive changes across the entire lifespan in patients with BD. In a cross-sectional study of patients with BD age 18–87, patients with BD and controls differed on multiple cognitive domains with only age predicting poorer performance on a measure of attention and speed, the Trail Making Test-Part A (Lewandowski, Sperry, Malloy, & Forester, 2014). A larger cross-sectional study concluded that cognitive performance declined in parallel in younger adults with and without a mood disorder [major depressive disorder (MDD) or (BD)] (Gualtieri & Johnson, 2008). Although MDD and BD patients were not analyzed separately, cognitive performance declined sharply after age 65 in both groups, with a differential rate of decline in the domains of memory, attention, processing speed, and executive function (Gualtieri & Johnson, 2008). In a recent 20-year cohort study, 164 participants with MDD or BD with psychotic features experienced significant declines in most cognitive domains that were larger than expected due solely to normal aging in the domains of verbal knowledge, fluency, and abstraction-executive function (Fett et al., 2020). Recently a cross-sectional study of patients with BD showed an age-related selective decline in attention compared to controls (Montejo et al., 2022a). Given these contradictory findings, we need to enhance our understanding of cognitive function in BD and its trajectory across the lifespan independent of mood states.

Three conceptual models that have been proposed to understand the trajectory of cognition in patients with severe mental illness can be considered to understand the trajectory of cognition in patients with BD specifically. According to the early impairment model (also referred to as ‘early hit’), some neurodevelopmental processes create a gap in performance observed between younger persons with and without a mental disorder that either persists or even narrows with aging (possibly due to a survival effect or resolution of symptoms). According to the accelerated aging model, cognitive decline occurs in patients with severe mental disorders faster than what would be expected with normal aging, creating a gap in performance between persons with and without a mental disorder that widens as they age. Last, in the combined model, an early neurodevelopmental impairment” may become more accentuated with time through accelerated aging (Berk et al., 2017; Vieta et al., 2018).

To evaluate the relevance of these conceptual models to BD, we conducted a cross-sectional analysis comparing cognitive performance of participants with BD who were 18–86 years of age and non-psychiatric (‘healthy’) control participants (HC). We hypothesized that participants with BD would present with an early deficit (early impairment) and more rapid cognitive decline than HC (i.e. accelerated aging) (Depp et al., 2007; Gildengers et al., 2007).

## Methods

### Participants

Participants included in this analysis were recruited from four separate studies that took place at the Centre for Addiction and Mental Health (CAMH), Toronto, Ontario, Canada. CAMH is an academic hospital offering secondary and tertiary psychiatric care to patients in a large urban and suburban catchment area. Participants with BD were recruited based on referrals by their CAMH clinicians, a research registry, or response to advertisements posted in the hospital. Non-psychiatric HC participants were recruited through existing study databases and posted advertisements.

BD participants underwent a comprehensive clinical assessment to confirm diagnosis, rule out active substance use and other psychiatric disorders, and assess current mood symptoms. This assessment included the Structured Clinical Interview for DSM-IV Disorders (SCID-IV-TR) (American Psychiatric Association, 2002), the Young Mania Rating Scale (YMRS) (Young, Biggs, Ziegler, & Meyer, 1978), and the 17-item Hamilton Depression Rating Scale (HDRS) (Hamilton, 1960). Participants with BD had to meet the following criteria to be included in this analysis: diagnosis of BD I or II according to the DSM IV criteria; euthymia defined by the absence of significant (hypo)manic or depressive symptoms for at least four weeks before testing, as indicated by scores ⩽ 10 on both the YMRS and HDRS; absence of substance use disorder in the six months prior to participation; and negative urine drug screen result. In addition, they were excluded if they had any type of dementia or other neurological disorder affecting the central nervous system; a previous diagnosis of schizophrenia, schizoaffective or other psychotic disorders; and electroconvulsive therapy (ECT) within 6 months of assessment given the potential impact of ECT on cognition. Non-psychiatric HCs were excluded if they: (i) met DSM IV criteria for any Axis I psychiatric diagnosis; (ii) had used any psychotropic medication except for sedative-hypnotics during the last four weeks; (iii) had a history of head trauma resulting in loss of consciousness for more 30 min that required medical attention; (iv) had a diagnosis of an unstable physical illness (e.g. active cancer); or (v) had stroke or another significant neurological disorder within last year.

All four studies were carried out in accordance with ethical principles for medical research involving humans (Declaration of Helsinki); they were approved by the CAMH Research Ethics Board and all participants provided written informed consent prior to beginning any study procedures.

### Cognitive assessment

All participants completed a comprehensive neuropsychological battery that included the MATRICS Cognitive Consensus Battery (Bo et al., 2017; Nuechterlein et al., 2008) and additional tests to assess seven different cognitive domains: attention/vigilance [Continuous Performance Tests Identical Pairs (CPT-IP)]; information processing speed [Trail Making Test Part A (TMT-A); Brief Assessment of Cognition in Schizophrenia Symbol Coding (BACS-SC)]; executive function/working memory [Letter Number Span (LNS); Wechsler Memory Scale III Spatial Span (SS); Trail Making Test Part B (TMT-B); ratio of TMT-B over TMT-A; Stroop Neuropsychological Screening Test (Stroop Ratio)]; visuo-spatial memory [Brief Visuospatial Memory Test-Revised (BVMT-R) (total learning score)]; verbal memory [Hopkins Verbal Learning Test (HVLT-R) (total learning score)]; reasoning and problem solving [Neuropsychological Assessment Battery Mazes (Mazes)]; and social cognition [Mayer-Salovey-Caruso Emotional Intelligence Test (MSCEIT)]. Pre-Morbid IQ was estimated with the Wechsler Test of Adult Reading (WTAR). See online Supplementary Table S1 for a brief description of how each of the tests was administered and scored.

### Statistical analyses

For demographic and clinical variables, group differences were evaluated with one-way analysis of variance (ANOVA) for continuous measures followed by pair-wise comparisons with Bonferroni corrections, and χ^2^ tests for categorical measures followed by inspection of likelihood ratio χ^2^ contributions.

Cognitive scores were assessed for normality with tests of skewness and kurtosis; for the BACS-SC test, natural logarithm was used in the analysis to normalize the distribution of scores (however, values are reported in their original units). Each one of the 12 cognitive measures were analyzed with two different multiple regression models. In the first set of models, we compared four groups: younger BD (age ⩽49 years; BD-Y), older BD (⩾age 50 years; BD-O), younger comparators (HC-Y), and older comparators (HC-O). In the second set of models, we compared BD and HC using age as a continuous independent variable and tested for the interaction between the two diagnostic groups (i.e. BD *v.* HC) and age. In the first set of models, the analyses were adjusted for pre-morbid IQ scores, years of education, YMRS and HDRS scores, history of smoking or psychosis, number of depressive episodes and psychiatric hospitalizations. The second set of models adjusted for age and the same covariates. These covariates were selected because they were the demographic and clinical variables that differed statistically between at least two of the four groups, with the addition of the YMRS and HDRS scores. However, since duration of illness and age of onset of BD were correlated to age (*r* = 0.78 and *r* = 0.57, respectively), they were not used as covariates to avoid overfitting. To assess the significance of each set of models, we used a Bonferroni correction to control for multiple comparisons; thus, a model was considered significant only if the *p* of the model was < 0.004 (i.e. 0.05/12). When a model was statistically significant, we analyzed the differences among groups by carrying out post-hoc analyses employing Sidak post-tests. Finally, effect sizes (Cohen's *d*) were calculated to assess the magnitude of the differences between groups. All analyses were performed using STATA Version 13.

## Results

The sample consisted of 315 participants: 118 with BD and 197 HC. Characteristics of the sample are shown in Table 1. The BD and HC groups were not different statistically in terms of age and sex. However, they differed on several other variables (see Table 1). Similarly, the four age groups differed on several variables. In particular, compared to the BD-O participants, the BD-Y participants were more likely to have a history of smoking or psychotic symptoms during episodes, and had a lower number of depressive episodes or psychiatric hospitalizations. The BD-Y group also had lower pre-morbid IQ than the BD-O group.

**Table 1.** Demographic and clinical characteristics

Characteristic	Bipolar disorder young (BD-Y) *N* = 70	Control young (HC-Y) *N* = 153	Bipolar disorder old (BD-O) *N* = 48	Control old (HC-O) *N* = 44	*F* or Pearson statistic	All groups *p* value (*p* < 0.05)	BD-*All N* = 118	HC-*All N* = 197	*F* or Pearson statistic	BD-*All* v. HC-*All p* value (*p* < 0.05)
Female sex^1^	43 (61.4)	78 (50.9)	30 (62.5)	24 (54.5)	3.23 (3–315)	0.360	73 (61.9)	102 (51.8)	3.04 (1–315)	0.081
Age^2^	27.36 ± 6.21	28.12 ± 8.00	64.37 ± 9.81	65.97 ± 10.40	434.51 (3–311)	**<0.001^B,C^**	42.41 ± 19.87	36.57 ± 17.98	7.19 (1–313)	**0.008**
Years of education^2^	14.77 ± 1.85	15.63 ± 1.82	15.10 ± 2.54	14.91 ± 2.42	3.74 (3–311)	**0.012^Y^**	14.90 ± 2.15	15.49 ± 2.00	5.88 (1–313)	**0.016**
Age of onset of the disorder^2^	19.29 ± 5.45	NA	30.46 ± 16.07	NA	29.05 (1–116)	**<0.001^B^**	23.83 ± 12.31	NA	NA	NA
Length of illness in years 2	8.09 ± 5.97	NA	33.92 ± 14.38	NA	180.86 (1–116)	**<0.001^B^**	19.00 ± 16.52	NA	NA	NA
Lifetime history of smoking^1^	43 (61.4)	18 (32.7)	25 (55.6)	9 (23.7)	19.89 (3–208)	**<0.001^Y,O^**	68 (59.1)	27 (29.0)	18.78 (1–208)	**<0.001**
Number of manic episodes^2^	2.50 ± 4.18	NA	4.02 ± 6.04	NA	2.33 (1–104)	0.130	3.19 ± 5.13	NA	NA	NA
Number of depressive episodes^2^	6.22 ± 7.95	NA	12.19 ± 18.73	NA	5.61 (1–115)	**0.019^B^**	8.63 ± 13.61	NA	NA	NA
YMRS total score^2^	1.80 ± 2.11	NA	1.83 ± 2.68	NA	0.01 (1–116)	0.939	1.81 ± 2.34	NA	NA	NA
HDRS total score^2^	4.19 ± 2.62	NA	3.42 ± 3.03	NA	2.16 (1–116)	0.144	3.87 ± 2.81	NA	NA	NA
History of psychosis^1^	49 (70.0)	NA	17 (35.4)	NA	13.82 (1–118)	**<0.001^B^**	66 (55.9)	NA	NA	NA
History of suicide attempt^1^	13 (18.6)	NA	14 (29.17)	NA	1.81 (1–118)	0.178	27 (22.9)	NA	NA	NA
History of psychiatric hospitalization^1^	53 (75.7)	NA	39 (81.3)	NA	0.51 (1–118)	0.476	92 (78.0)	NA	NA	NA
Number of psychiatric hospitalizations^2^	1.33 ± 1.24	NA	5.31 ± 8.55	NA	14.81 (1–116)	**<0.001^B^**	2.95	NA	NA	NA
Wechsler Test of Adult Reading (WTAR)^2^	41.97 ± 6.11	42.73 ± 5.19	44.75 ± 5.15	45.02 ± 4.55	4.25 (3–213)	**0.006^B^**	43.11 ± 5.88	43.74 ± 5.03	0.7 (1–215)	0.402
Mini-Mental State Examination^2^	29.26 ± 0.96	29.48 ± 0.78	28.70 ± 1.35	29.14 ± 1.02	7.97 (3–303)	**<0.001^B^**	29.03 ± 1.17	29.40 ± 0.85	10.00 (1–305)	**0.002**

BD-Y, bipolar disorder –young group; HC-Y, healthy control –young group; BD-O, bipolar disorder –old group; HC-O, healthy control –old group; d.f., degrees of freedom; YMRS, Young Mania Rating Scale; HDRS, Hamilton Depression Rating Scale; NA, not applicable.

1 Values shown as: *n* (%). Pearson Chi-square test, Pearson value reported with d.f. and sample size in parenthesis.

2 Values shown as mean ± standard deviation. One-way ANOVA, *F* statistic reported with the between-group and the within group d.f. in parenthesis.

^Y^Young BD statistically different from young control.

^O^Old BD statistically different from old control.

^B^Old BD statistically different from young BD.

^C^Old control statistically different from young control.

**Fig. 1.**
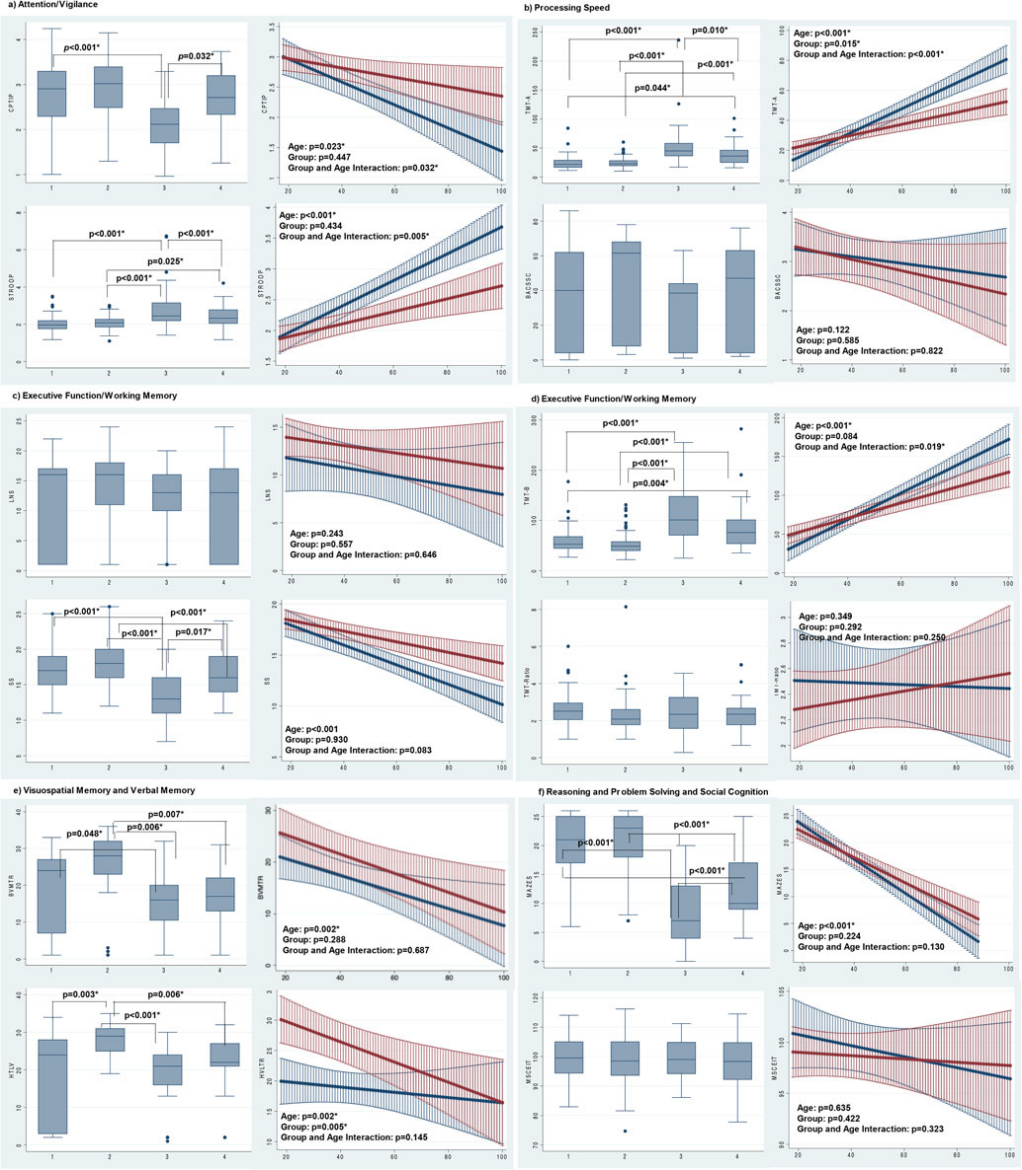
Cognitive performance across the life-span. Bar Graphs (Left): Multiple linear regression adjusted for years of education, Young Mania Rating Scale (YMRS) and Hamilton Depression Rating Scale (HDRS) scores, lifetime number of depressive episodes, lifetime history of psychosis, and lifetime history of smoking. A multiple linear regression was statistically significant if its p-value were <0.004 (Bonferroni correction, p< 0.05/12). When the p-value of the model was <0.004, we proceed with Sidak post-test to ascertain where the difference between the groups was found. The p-values above the lines refers to the Sidak post-test; the lines without a p-value denote lack of statistical significance after the Sidak post-test. 1=BD-Y, 2=HC=Y, 3=BD-O, 4=HC-O Linear Regression Graphs (Right): Regression models with cognitive measures as dependent variables and the above covariates including age. The interaction between age in years and diagnosis (bipolar disorder vs. comparator) was analyzed in a full factorial. *Stroop represents executive function.

Table 2 presents the results of the 12 cognitive tests, the comparison of the four groups according to the first set of models, and the effect sizes of the relevant pairwise groupings. Figure 1 presents the same data graphically; it uses bar graphs for the first set of models (i.e. the models based on four groups categorized according to age and diagnosis). The results of the second set of models shows the regression lines for the BD and HC groups with their 95% confidence intervals (CIs).

**Table 2.** Results of cognitive tests in participants with BD and healthy control participants

Cognitive test	Bipolar disorder *young (BD-Y)N* = 70	Control *young (HC-Y) N* = 153	Bipolar disorder *old (BD-O) N* = 48	Control *old (HC-O) N* = 44	F statistic *p* value (*p* < 0.004)^1^	*Effect Size d* (*95%CI*)		
						Bipolar disorder young v. young control (Y)	Bipolar disorder old v. old control (O)	Bipolar disorder old v. Bipolar disorder young (B)
Attention/Vigilance								
Continuous Performance Tests Identical Pairs (CPT-IP)	2.79 ± 0.69	2.93 ± 0.64	2.10 ± 0.60	2.67 ± 0.66	5.10 (11–204), **<0.001^O,B^**	−0.21 (−0.52 to 0.10)	−0.91 (−1.47 to −0.35)	−1.04 (−1.49 to −0.58)
Processing Speed								
Trail Making Test A (TMT-A)	24.32 ± 11.25	24.29 ± 8.13	51.80 ± 33.89	37.92 ± 17.95	12.34 (11–293), **<0.001^O,B,C^**	0.003 (−0.28 to 0.29)	0.51 (0.08–0.92)	1.18 (0.78–1.58)
BACS Symbol Coding (BACS-SC)	34.59 ± 29.72	47.15 ± 29.22	30.10 ± 19.08	36.65 ± 28.23	1.65 (11–154), 0.089^None^	−0.43 (−0.80 to 0.05)	−0.28 (−0.83 to 0.28)	−0.17 (−0.60 to 0.27)
Executive Function/Working Memory								
Letter Number Span (LNS)	11.93 ± 7.30	12.99 ± 7.28	11.31 ± 5.45	11.72 ± 7.41	1.61 (11–256), 0.097^None^	−0.15 (−0.43 to 0.14)	−0.06 (−0.60 to 0.47)	−0.09 (−0.52 to 0.34)
WMS-III Spatial Span (SS)	17.06 ± 2.83	18.28 ± 3.14	13.21 ± 3.34	16.25 ± 2.95	**10.35 (11–243), <0.001^O,B,C^**	−0.40 (−0.72 to 0.09)	−0.96 (−1.39 to −0.52)	−1.26 (−1.66 to −0.86)
Trail Making Test B (TMT-B)	58.55 ± 23.42	52.79 ± 21.09	115.60 ± 56.15	85.87 ± 45.67	**13.36 (11–243), <0.001^B,C^**	0.26 (−0.05 to 0.57)	0.58 (0.15–1.0)	1.43 (1.01–1.84)
Trail Making Test Ratio	2.55 ± 0.88	2.28 ± 0.89	2.50 ± 1.05	2.38 ± 0.87	2.57 (11–244), 0.004^None^	0.31 (−0.01 to 0.61)	0.12 (−0.29 to 0.53)	−0.05 (−0.42 to 0.32)
Stroop Neuropsychological Screening Test (Stroop Ratio)	2.02 ± 0.42	2.06 ± 0.33	2.81 ± 1.11	2.39 ± 0.62	6.36 (11–241), **<0.001^O,B,C^**	−0.10 (−0.41 to 0.20)	−0.46 (−0.03 to 0.88)	1.01 (0.62–1.40)
Visuospatial Memory								
Brief Visuospatial Memory Test-Revised (BVMT-R)	18.84 ± 10.78	24.63 ± 10.75	14.57 ± 8.09	16.88 ± 8.50	**3.03 (11–154), 0.001^B,C^**	−0.54 (−0.92 to 0.15)	−0.28 (−0.82 to 0.16)	−0.42 (−0.86 to 0.02)
Verbal Memory								
Hopkins Verbal Learning Test - Revised (HVLT-R)	20.07 ± 11.43	28.22 ± 3.97	18.37 ± 8.39	21.16 ± 8.61	**3.62 (11–162), <0.0001^Y,C^**	−0.90 (−1.28 to −0.51)	−0.33 (−0.86 to 0.21)	−0.16 (−0.59 to 0.27)
Reasoning and Problem Solving								
NAB Mazes (Mazes)	19.72 ± 5.53	21.35 ± 4.79	8.53 ± 5.80	12.16 ± 5.53	**16.13 (11–206), <0.001^B,C^**	−0.32 (−0.63 to 0.005)	−0.63 (−1.18 to −0.09)	−1.99 (−2.50 to −1.48)
Social Cognition								
MSCEIT	99.83 ± 7.00	98.92 ± 8.02	99.32 ± 6.14	98.56 ± 8.50	2.05 (11–204), 0.025^None^	0.12 (−0.19 to 0.43)	0.10 (−0.42 to 0.63)	−0.08 (−0.50 to 0.35)

All values are presented as mean ± standard deviation; higher scores denote higher performance except for the Stroop and the Trail Making Tests.

BACS-SC, Brief Assessment of Cognition in Schizophrenia Symbol-Coding (total number of correct responses); BVM-TR, Brief Visuospatial Memory Test Revised (total recall score over three learning trials); CI, confidence interval; CPT-IP, Continuous Performance Test Identical Pairs (mean d’ value across 2-, 3-, and 4-digit conditions); HVLTR, Hopkins Verbal Learning Teste Revised (total number of words recalled correctly over three learning trials); LNS, Letter Number Span (number of correct trials); MSCEIT, Mayer-Salovey-Caruso Emotional Intelligence Test (branch standard score using general consensus scoring); NAB Mazes, Neuropsychological Assessment Battery Mazes (total raw score); SS, Spatial Span (sum of raw scores on forward and backward conditions); TMT-A, Trails Making A (time to completion – seconds); TMT-B, Trails Making B (time to completion – seconds); TMT-Ratio, Trails Making B/ Trails Making A ratio; Stroop, Stroop Neuropsychological Screening Test.

1 One-way ANCOVA controlling for years of education; HDRS and YMRS scores; history of smoking or psychosis; and number of depressive episodes or psychiatric hospitalizations.

Post-hoc comparisons with Bonferroni correction:.

^Y^Young BD statistically different from Young Control (1 v. 2).

^O^Old BD statistically different from Old Control (3 v. 4).

^B^Old BD statistically different from Young BD (1 v. 3).

^C^Old Control statistically different from Young Control (2 v. 4).

^None^No significant differences in any of the four post-hoc comparisons.

For these effect size calculations, the HC group was used as the reference group for the BD-Y and HC-Y pair and the BD-O and HC-O pair; the younger group was used as the reference for the BD-Y and BD-O pair and the HC-Y and HC-O pair.

**Fig. 2.**
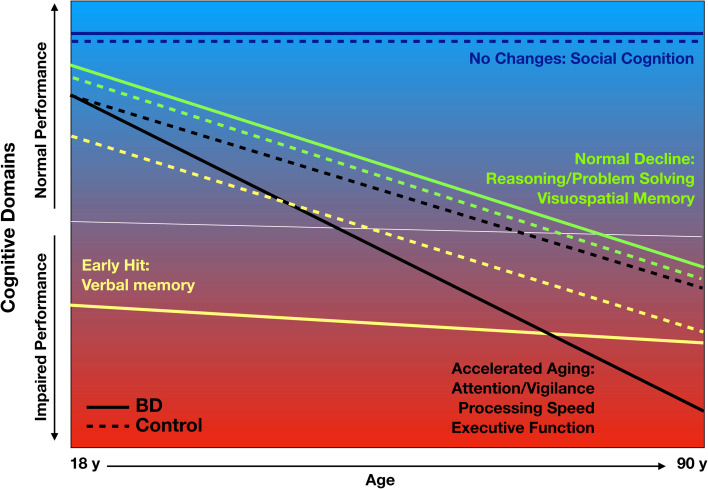
Theoretical model of cognitive function across the lifespan. The figure represents the theoretical model of cognitive function across the life span. The dashed lines represent controls and the solid lines represent BD.

Figure 2 summarizes all the cognitive trajectories graphically according to the early impairment, accelerated aging, and combined models. Trajectories for attention/vigilance, processing speed (as measured by the TMT-A), executive function/working memory (as measured by the SS and TMT-B) were all congruent with a pattern of accelerated aging with participants with BD showing a steeper decrease in performance than HC (Figs 1a–1d). By contrast, trajectories for visual spatial memory or reasoning/problem solving were consistent with normal aging (Figs 1e, 1f). The trajectory for verbal memory was consistent with an early impairment followed by normal aging, with participants with BD showing earlier deficits and then participants with HC, but not those with BD, showing a decrease in performance associated with aging (Fig. 1e). Finally, there were no differences in social cognition associated with age or diagnostic group (Fig. 1f).

## Discussion

BD is a severe mental illness associated with persistent cognitive impairments affecting multiple cognitive domains (Bourne et al., 2013). We found evidence supporting an accelerated-aging model for three cognitive domains (i.e. attention/vigilance, processing speed, and executive function/working memory) and an early-impairment model for one domain (verbal memory). Both patients and controls exhibited similar performance and age-related decline in visual spatial memory and reasoning/problem solving, with no differences or changes in social cognition. We found no evidence of a combination of the two models (i.e. an early impairment followed by accelerated aging) for any cognitive domain. The cognitive differences between various groups remained present when controlling for key factors such as cognitive reserve reflected by pre-morbid IQ or years of education, markers of severity of BD (i.e. history of psychosis, number of depressive episodes or psychiatric hospitalizations), and residual depressive or manic symptoms, all of which are known to affect cognitive performance.

There has been conflicting evidence in the literature as to whether BD constitutes a neuroprogressive disorder with early impairment in cognition and further deterioration associated with aging. Previous studies have suggested that some patients with BD exhibit broad impairment in cognition evident early and throughout their life although the results of these studies have been highly heterogeneous (Gildengers et al., 2009; Seelye et al., 2019). However, most longitudinal studies do not show a deterioration in cognition over periods of 1–5 years (Bora & Özerdem, 2017). This may be due to their relative short study duration as cohort studies with longer follow-up have reported differential changes in cognitive function compared to controls: one study with a follow-up of 9 years showed a difference between the cognitive trajectories of patients with BD and controls (Bora & Özerdem, 2017); in a 20-year cohort study, 164 patients with either MDD or BD with psychotic features experienced larger reduction in some cognitive domains than would be expected solely due to aging (Fett et al., 2020). Most other existing studies are limited by their shorter-term follow-up and small number of older adults. Our cross-sectional study, comparing younger and older groups of patients with BD or HC, suggests a more complex picture with an early impairment for some domains, accelerated aging for other domains, and normal aging for still other domains. Taken together, our findings and previous studies suggest that long follow-up (e.g. 20–30 + years) would be needed to detect differences in the trajectory in cognition of patients with BD and HC.

Our study has both strengths and limitations. Only a few studies have examined cognition in late-life BD including difference in cognitive profile across the lifespan. We used a comprehensive neurocognitive battery and all patients with BD were euthymic and stable for at least four weeks. Previous comparisons of younger and older patients with BD may have inflated age-related differences in cognitive performance because older patients are more likely to have residual mood symptoms that can be associated with acute cognitive dysfunction. The same reason, i.e. assessing euthymic patients, may explain the absence of differences in social cognition as a recent report suggests social cognition deficits in individuals with BD may be prevalent only during acute mood episodes (Kuo et al., 2021). In addition, while the MSCEIT has been extensively used in patients with BD, it may be limited in assessing broader social-cognitive domains relevant to BD, which could have contributed to our absence of finding related to social cognition (Eack et al., 2010; Gillissie et al., 2022; van Rheenen & Rossell, 2014).

One of the main limitations of our study was the fact that all participants with BD were receiving pharmacological treatment. Mood stabilizers, antipsychotic medications, anticholinergic agents, antidepressants, and sedative-hypnotics may significantly impair cognitive performance (Chew et al., 2008). For example, anticholinergic agents have been associated with increased brain atrophy and dysfunction in adults (Risacher et al., 2016). There is also evidence that medications such as lithium have neuroprotective effects (Diniz, Machado-Vieira, & Forlenza, 2013). Thus, we cannot ascertain the extent to which the cognitive differences we observed were due to psychotropic medications. Another major limitation is that this is a cross-sectional study with a moderate sample size. Therefore, it is possible that some of our findings are due to a survivor effect, according to which older patients with BD represent a subgroup of younger patients with BD who did not die prematurely because of a variety of factors related to their cognitive function. This is supported by a higher pre-morbid IQ and older age of onset in the BD-O group than the BD-Y group. However, observed cognitive differences between the two groups remained significant when we controlled for pre-morbid IQ. Also, despite our moderate sample size, we were able to detect several significant differences related to age or diagnosis (i.e. BD *v.* HC). While limited in our ability to infer causation, our results provide insight into cognitive trajectories in adults with BD, emphasizing the potential value of future larger cross-sectional studies across the lifespan given the challenges of recruiting a large sample of patients with BD and retaining and following them for more than 20 years.

## Conclusions

Our study supports the concept of accelerated aging in BD for the domains of attention/vigilance, processing speed, and executive function/working memory. It also supports the concept of an early impairment associated with some (unspecified) neurodevelopmental process for verbal memory, and declines due to normal aging for the domains of reasoning/problem solving and visuospatial memory. Both larger cross-sectional studies and longitudinal studies are needed to explore trajectories of cognitive domain performance in BD. These future studies should also assess biomarkers to provide insight into the mechanisms underlying cognitive dysfunction and decline in BD and to identify targets for novel interventions.

## Supplementary material

For supplementary material accompanying this paper visit https://doi.org/10.1017/S0033291722003622. click here to view supplementary material
