# Supplementary material for: A cross-sectional study of cognitive performance in bipolar disorder across the lifespan: the cog-BD project
Source: Psychol Med. 2022 Dec 5;53(13):6316–24. doi: 10.1017/S0033291722003622 (PMC10520592; doi:10.1017/S0033291722003622)
Supplement: Supplementary file 1 [file S0033291722003622sup001.docx]

**Supplement Table 1.**

**Table 1.** Description of cognitive tests

| **Test** | **Description** |
| --- | --- |
| MATRICS Consensus Cognitive Battery (MCCB) | |
| Brief Assessment of Cognition in Schizophrenia – Symbol Coding (BACS-SC) | Participants were presented with a legend containing unique symbols and digits (1-9) as well as a table with symbols on top and blank cells beneath. After a practice trial, they were asked to record the corresponding number beneath each symbol. Scoring was based on the total number of correct responses within the 90-second limit. Higher scores denote higher performance. |
| Brief Visuospatial Memory Test – Revised (BVMT-R) | Participants were presented with a display of six geometric figures for 10 seconds, after which they were asked to reproduce the figures on a blank page. Scoring was based on drawing accuracy and placement of the figures across three trials. Higher scores denote higher performance. |
| Continuous Performance Test – Identical Pairs (CPT-IP) | Participants were asked to pay close attention to numbers quickly flashing on the screen and to click the left mouse button when identical digits flashed consecutively. Scoring was based on the mean of the d-prime values for the 2-, 3-, and 4-digit trials obtained though the CPT-IP program. Higher scores denote higher performance. |
| Hopkins Verbal Learning Test – Revised (HVLT-R) | The administrator read a 12-word list containing three semantic categories after which participants were asked to recall the words. The total score is based on free recall across the three administered trials. Higher scores denote higher performance. |
| Letter Number Span (LNS) | The administrator read aloud sequences of letters and numbers. Participants were asked to mentally separate and reorder the strings numerically and then alphabetically before repeating them back to the administrator. Scoring was based on the number of correct responses across the administered trials. Higher scores denote higher performance. |
| Mayer-Salovey-Caruso Emotional Intelligence Test: Managing Emotions (MSCEIT) | In this paper-and-pencil ability test, participants were asked to read scenarios about people in emotion-laden situations and evaluate the effectiveness of alternative courses of action. Two sub-tests make up the test: social and emotion management. Standard scores were obtained using MSCEIT Branch 4 Scoring Program. Higher scores denote higher performance. |
| Neuropsychological Assessment Battery: Mazes (Mazes) | Participants completed paper-and-pencil mazes of increasing difficulty. Scores were assigned based on the time it took to complete each maze. The total score used in analyses was the sum of individual maze scores. Higher scores denote higher performance. |
| Trail Making Test A (TMT-A) | Participants were asked to draw lines to connect irregularly placed circles with numbers in a consecutive order. Participants had a time limit of 300 seconds. Scoring was based on the time per correct connection. Higher scores denote worse performance. |
| Wechsler Memory Scale-III Spatial Span (SS) | Participants tapped 10 irregularly spaced cubes after observing the administrator perform the task. Scoring was based on the total number of correctly performed forward and backward sequences. Higher scores denote higher performance. |
| Other Tests | |
| Stroop Neuropsychological Screening Test (Stroop) | Participants were first asked to read a list of 112 words (colour task) and then to name the ink colour of the printed words (colour-word task). Both tasks had a time-limit of 120 seconds. Interference ratio was based on time per correct response of the colour-word to colour task. Lower scores denote better performance. Higher scores denote worse performance. |
| Trail Making Test B (TMT-B) | Participants were asked to draw lines to connect irregularly placed circles containing numbers and letters in a consecutive order. Participants had a time limit of 300 seconds. Scoring was based on the time per correct connection. Higher scores denote worse performance. |
| Wechsler Test of Adult Reading (WTAR) | Participants were asked to read aloud 50 irregularly-spelled words of increasing difficulty. Raw score was based on the total number of correctly pronounced words; an age-based standard score was computed. Higher scores denote higher performance. |

MATRICS: Measurement And Treatement Research to Improve Cognition in Schizophrenia
